# Inferring Cetacean Population Densities from the Absolute Dynamic Topography of the Ocean in a Hierarchical Bayesian Framework

**DOI:** 10.1371/journal.pone.0120727

**Published:** 2015-03-18

**Authors:** Mario A. Pardo, Tim Gerrodette, Emilio Beier, Diane Gendron, Karin A. Forney, Susan J. Chivers, Jay Barlow, Daniel M. Palacios

**Affiliations:** 1 Posgrado en Ciencias del Mar y Limnología, Universidad Nacional Autónoma de México, Distrito Federal, 04510, Mexico; 2 Unidad La Paz, Centro de Investigación Científica y de Educación Superior de Ensenada, La Paz, Baja California Sur, 23050, Mexico; 3 Marine Mammal and Turtle Division, Southwest Fisheries Science Center, National Marine Fisheries Service, National Oceanic and Atmospheric Administration, La Jolla, California, 92037-1508, United States of America; 4 Centro Interdisciplinario de Ciencias Marinas, Instituto Politécnico Nacional, La Paz, Baja California Sur, 23096, Mexico; 5 Marine Mammal Institute, Oregon State University, Newport, Oregon, 97365, United States of America; University of Vigo, SPAIN

## Abstract

We inferred the population densities of blue whales (*Balaenoptera musculus*) and short-beaked common dolphins (*Delphinus delphis*) in the Northeast Pacific Ocean as functions of the water-column’s physical structure by implementing hierarchical models in a Bayesian framework. This approach allowed us to propagate the uncertainty of the field observations into the inference of species-habitat relationships and to generate spatially explicit population density predictions with reduced effects of sampling heterogeneity. Our hypothesis was that the large-scale spatial distributions of these two cetacean species respond primarily to ecological processes resulting from shoaling and outcropping of the pycnocline in regions of wind-forced upwelling and eddy-like circulation. Physically, these processes affect the thermodynamic balance of the water column, decreasing its volume and thus the height of the absolute dynamic topography (ADT). Biologically, they lead to elevated primary productivity and persistent aggregation of low-trophic-level prey. Unlike other remotely sensed variables, ADT provides information about the structure of the entire water column and it is also routinely measured at high spatial-temporal resolution by satellite altimeters with uniform global coverage. Our models provide spatially explicit population density predictions for both species, even in areas where the pycnocline shoals but does not outcrop (*e*.*g*. the Costa Rica Dome and the North Equatorial Countercurrent thermocline ridge). Interannual variations in distribution during El Niño anomalies suggest that the population density of both species decreases dramatically in the Equatorial Cold Tongue and the Costa Rica Dome, and that their distributions retract to particular areas that remain productive, such as the more oceanic waters in the central California Current System, the northern Gulf of California, the North Equatorial Countercurrent thermocline ridge, and the more southern portion of the Humboldt Current System. We posit that such reductions in available foraging habitats during climatic disturbances could incur high energetic costs on these populations, ultimately affecting individual fitness and survival.

## Introduction

Blue whales (*Balaenoptera musculus*) and short-beaked common dolphins (*Delphinus delphis*) feed primarily on low-trophic-level prey [1] and have high energetic requirements [2]. Both species numerically dominate the cetacean fauna in the most productive regions of the Northeast Pacific Ocean [2–4], despite having suffered the pressure of commercial whaling in the case of the blue whale [5–7], and bycatch in fisheries in the case of the short-beaked common dolphin [8,9]. Currently, better knowledge of the dynamics that drive their distribution is needed in order to evaluate potential threats and to implement future conservation measures [10,11]. Effective conservation policies for cetacean species, as with other groups of marine megafauna, should be based on the identification of critical habitats and on the characterization of the influence of extreme environmental changes, including the uncertainty associated with each process [12], in a way that their effect can be measured, monitored, and predicted. Additionally, approaches that include species-specific responses to changes in the oceanic environment would be useful for interpreting and predicting population trends in the context of climate change impacts [13].

During the summer-autumn of the Northern Hemisphere, blue whales forage at high latitudes in cold, well-mixed waters modified by upwelling [14], mainly in the California Current System [15,16], the Alaska Gyre, and the Aleutian Islands [17,18]. In winter-spring, they migrate to breed and feed in lower latitudes, especially in the Gulf of California [19] and the Costa Rica Dome. During this migration the species also uses other transitory areas to feed, such the Frontal System off Baja California [20]. Blue whales from the Southern Hemisphere forage in the southern Humboldt Current System and the Antarctic Circumpolar Current in summer-autumn [21]. Some of them migrate in winter-spring to the northern Humboldt Current System [21,22], and the Equatorial Cold Tongue [23] for breeding. Although blue whales have been recorded year-round at the Costa Rica Dome, it is not clear whether those animals come from both hemispheres, or if at least some belong to a resident population [24]. In contrast, short-beaked common dolphins do not have an evident seasonal migration pattern and are distributed year-round in equatorial and subtropical waters characterized by a shallow but weak thermocline [25,26].

The cetacean survey dataset we used in this study has an extensive coverage of the Northeast Pacific Ocean and is the largest in the world [27]. These data have allowed for the estimation of regional and local abundances of various cetacean species [4,28–30], but the traditional distance sampling analysis [31,32] does not permit addressing trends of population densities explicitly in space and time. Alternative approaches have been adopted to predict spatially explicit distributions of species based on their affinity to particular environmental conditions measured concurrently, either *in situ* or using remotely sensed variables. Such studies have successfully captured the patterns of spatial distribution for several species in the eastern tropical Pacific [33,34] and in the California Current System [33,35]. Although some environmental variables collected *in situ*, such as the mixed layer depth or the pycnocline depth, may contain direct information on water-column dynamics, their sparse coverage in space and time limit them as predictors of long-term, broad-scale spatial patterns.

While most oceanographic variables obtained from remote sensing measurements typically cover large spatial extents, they represent only a small portion of the vertical habitat, usually from the surface to the first 10 m of the water column. Yet, it is well known that the distribution of marine megafauna, including cetaceans, is greatly influenced by the physical structure of the entire water column, especially in terms of the vertical displacements of the pycnocline, which in some areas may reach the surface (*i*.*e*. “outcrop”) [36,37]. In the open ocean, the pycnocline separates the water column into a warmer, less dense upper layer, and a colder, denser, and nutrient-enriched deeper layer. The pycnocline boundary can deepen, shoal, or even outcrop due to a variety of forces. The most productive ecosystems in the Northeast Pacific Ocean occur in regions of pycnocline shoaling and outcropping, as they are forced by intense wind stress and eddy-like circulation [38,39]. In these regions, new nutrients enrich the euphotic zone, producing large phytoplankton blooms [40–42] that aggregate low-trophic-level prey, such as krill (Crustacea: Euphausiacea) and small pelagic fish (*i*.*e*. sardine and anchovy) [14,43–49], which represent nearly 100% and ~60% of the diets of blue whales [1,14] and short-beaked common dolphins [1,50], respectively.

**Fig 1:**
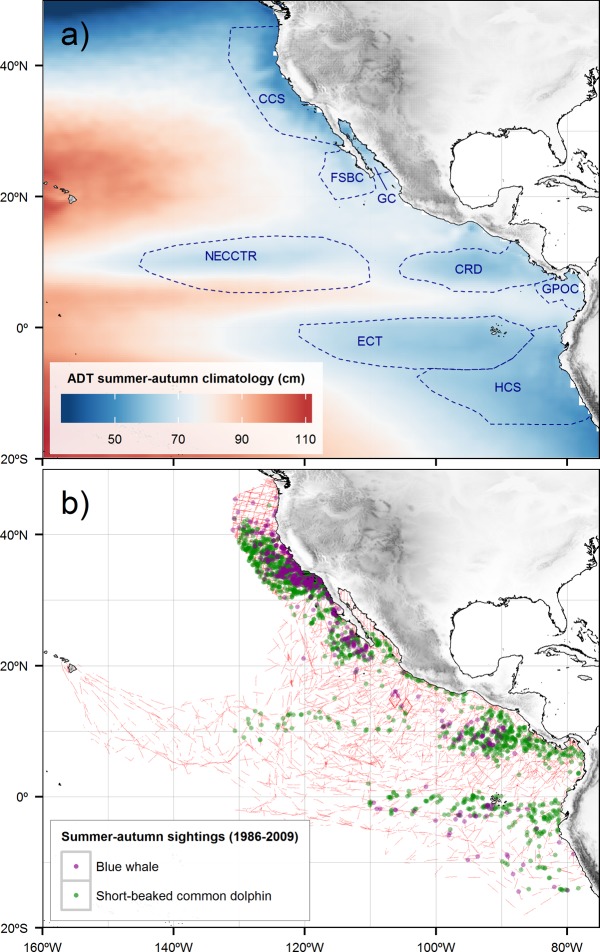
Study area, survey effort, and sightings. A) Main features of low absolute dynamic topography (ADT) in the Northeast Pacific Ocean: the California Current System (CCS), the Frontal System off Baja California (FSBC), the Gulf of California (GC), the North Equatorial Countercurrent thermocline ridge (NECCTR), the Costa Rica Dome (CRD), the Gulf of Panama and off Colombia (GPOC), the Equatorial Cold Tongue (ECT), and the Humboldt Current System (HCS). B) Blue whale and short-beaked common dolphin sightings (dots colored), and survey effort (thin red lines) across the Northeast Pacific Ocean, collected during summer-autumn (July-December) at 18 yearly surveys, spanning the period 1986–2009.

At the scale of an ocean basin, shoaling and outcropping of the pycnocline reduce the amount of warmer and less dense water in the water column, decreasing the total volume and thus the height of the absolute dynamic topography (ADT) of the ocean’s surface [51–53]. At a given location, this elevation is calculated by summing the sea level anomalies (SLA), which correspond to the deviations from the historical mean of the sea surface height (SSH) [54], plus the mean dynamic topography, which is the part of mean SSH due to permanent currents (that is, the mean SSH minus a geoid of reference). In regions with a deep pycnocline, there is more warm water in the upper layer, which increases the volume and the total height of the water column. Thus, pycnocline shoaling decreases the ADT, whereas pycnocline deepening increases it [52,55]. In mid-latitude regions, wind-curl-forced Ekman pumping causes the pycnocline to outcrop, such that the two-layer configuration described above breaks down [55]. However, since in that case cold water occupies a large portion of the mixed upper water column, the ultimate result is also a decrease in volume, and thus in the ADT. Therefore, low ADTs reveal the physical structure of the most productive habitats throughout the Northeast Pacific Ocean (Fig. 1), regardless of the surface or subsurface phenomena forcing them because this measurement includes information on both permanent currents as well as mesoscale phenomena [56,57]. Given this context, remotely sensed ADT is a variable particularly well-suited to infer the distribution of blue whale and short-beaked common dolphin population densities, especially in the northeastern tropical Pacific, where other surface variables do not consistently capture subsurface features such as the Costa Rica Dome or the North Equatorial Countercurrent thermocline ridge. The advantage of using ADT over other surface variables has already been demonstrated in a coastal wind-forced upwelling area for inferring the spawning of low-trophic-level fish [58].

When eddy-like circulation forces the pycnocline upwards, the latter does not typically outcrop, but only shoals to a certain depth [59,60], enough in some cases to reach the euphotic zone, where nutrients can be taken up by phytoplankton [61]. The effects of this pycnocline shoaling cannot always be inferred from changes in surface conditions such as temperature or chlorophyll-*a* concentration [62,63]. The Costa Rica Dome (Fig. 1) features this type of eddy-like forcing [59,62] and sustains blue whales and short-beaked common dolphins year-round [24,36]. The North Equatorial Countercurrent thermocline ridge (Fig. 1) [60,62] is also inhabited year-round by short-beaked common dolphins [25,64], while blue whales have been reported there occasionally [36,65,66]. The force acting on the upper layer in the mid latitudes is different. There, upwelling is usually driven by strong coastal winds that produce pycnocline outcropping, instead of just shoaling [67]. As a consequence, the upper water column mixes, making new nutrients available over a thicker layer and allowing the phytoplankton to distribute itself more evenly in the euphotic zone [38]. In these regions, the processes of cold water intrusion and elevated biological production are readily detected by traditional surface variables such as sea surface temperature and chlorophyll-*a* concentration [68], which can be measured remotely or *in situ*. The California Current and the Humboldt Current are typical mid-latitude upwelling systems that sustain the highest biological productions in the eastern Pacific (Fig. 1) [69,70].

Our goal was to infer the population densities of blue whales and short-beaked common dolphins in the Northeast Pacific Ocean from the remotely sensed ADT, based on the hypothesis that their distributions in space and time respond to the availability of low-trophic-level prey, which, in turn, is abundant in regions of pycnocline shoaling and outcropping. Despite the disparity that exists between a migrating and a non-migrating species in terms of habitat use, we expected that their relationship with the physical structure of the water column would be similar because of their need to feed constantly on low-trophic-level prey, regardless of the season, and because we were only evaluating the portion of the blue whale migration occurring in the summer-autumn period. Therefore, potential changes in the habitat requirements of this species during their winter-spring migration period were not included in the analyses of this study (see Materials and Methods below).

Since El Niño-Southern Oscillation (ENSO) anomalies modify considerably the pycnocline structure in the eastern Pacific [71], we expected to infer the response of both species to these anomalies in terms of inter-annual re-distributions of population density. We chose a hierarchical modeling approach [72,73] because it allowed for the incorporation of the observational stochastic processes that generated the data (*i*.*e*. species detectability and the areas effectively sampled) as well as the ecological processes (*i*.*e*. the response of the species’ distribution to the physical structure of the habitat) as connected sub-models [13,72,74]. The analysis of these models was carried out in a Bayesian framework, which propagated the uncertainty related to those separate processes in a two-way feedback among sub-model parameters. Also, the Bayesian philosophy acknowledges the non-experimental nature of ecological studies, allows for the inclusion of previous knowledge on the parameters, and provides their estimations in terms of probabilities, instead of fixed quantities [75–79]. This type of approach has served recently to estimate trends in abundance of various cetacean species with improved accuracy [80–83], but this is the first time it is used for the inference of their spatial distribution.

## Materials and Methods

The fieldwork was carried out with permits under the US Marine Mammal Protection Act and the Endangered Species Act, issued to the Marine Mammal and Turtle Division of the Southwest Fisheries Science Center (SWFSC), La Jolla, California. Research clearances from the governments of Peru, Ecuador, Colombia, Panama, Nicaragua, El Salvador, Guatemala, Honduras, Costa Rica, France, and Mexico were obtained to collect data in the Exclusive Economic Zone waters of each country for each year of the eastern tropical Pacific surveys. Line-transect effort and cetacean sightings were used to estimate the population densities of blue whales and short-beaked common dolphins, following distance sampling techniques [31,32]. The data were collected during 19 annual ecosystem surveys made across the Northeast Pacific Ocean by the SWFSC during the summer-autumn in the Northern Hemisphere (July-December), spanning the period 1986–2009 (Fig. 1; S1 Fig.). The survey followed *zig-zag* arrangements of transects designed to evenly cover specific strata [84]. The effectively sampled strip half-width along the track lines was inferred for each species as a function of the perpendicular distance between the groups of animals and transects. We used Beaufort sea state and the size of the groups as the most important covariates affecting the detection distance [85–87]. This allowed us to estimate the area effectively sampled, and thus, the population density in individuals per unit of area (ind. km^-2^).

The ADT variable was produced by the *Segment Sol multimissions d'ALTimétrie*, *d'Orbitographie et de localisation precise* and the Data Unification and Altimeter Combination System in France. Access to ADT weekly means at a spatial resolution of 1/3 x 1/3-degree cells, spanning the period 1992–2013, was granted by the Archiving, Validation, and Interpretation of Satellite Oceanographic Data program (AVISO; http://www.aviso.oceanobs.com/en/). All the daily survey effort and sightings data were gridded into cells of the same size as the ADT’s spatial resolution. This produced a series of very short transects inside each cell (~22 km), which helped to reduce the variance due to the spatial heterogeneity of the sampling effort [88], and to meet the assumption that the probability of group counts in the transects was Poisson-distributed by reducing the variance of encounters among cells (see model description below). Each cell surveyed had a corresponding weekly value of ADT. S1 Fig. shows the frequency distribution of the main variables used in our models.

The hierarchical models, comprising the observational and the ecological processes, were written in an algebraically explicit manner using the OpenBUGS software (http://www.openbugs.info/) (S1 Text), which is an implementation of the BUGS language [89,90] that performs Bayesian inference using the Gibbs sampler algorithm [91,92] to sample from the posterior distributions of the parameters through a Markov Chain Monte Carlo procedure. We ran 10,000 iterations with a burn-in phase of 2,000 samples, and retained every tenth value, for a posterior sample of 800. Priors for all the stochastic parameters were non-informative, except for the probability of detecting the species on the track line (see description below). The package R2WinBUGS [93] was used as interface with OpenBUGS to perform all the analyses from R [94]. The observational process began by defining the likelihood of the perpendicular distances (*x*) from the transect line to the groups, collected at each sighting (*j*). This was based on the assumption that the probability of detecting the species at sea decreased when the animals were far from the observers [31,32]. Conditional on the sighting covariates, distances were assumed to be half-normally distributed:

$$x_{j}\sim N\left(0,\sigma^{2}{}_{j}\right)$$ (1)

The standard deviation of this likelihood defined the effective strip half-width (*w*) at which the groups of animals were effectively sampled [95]:

$$\sigma_{j}=w_{j}\;\sqrt{\frac{2}{\pi }}$$ (2)

This width *w* may increase or decrease exponentially depending on covariates that might affect the detectability of animals [96]. We chose the Beaufort sea-state (*b*) because it has been reported as one of the most important external conditions affecting the detectability of cetaceans [85,95]. Sea state was recorded on all effort segments and for each sighting. We also used the group size (*s*) as a covariate because large groups are expected to be more detectable than small groups [85]. Group sizes for blue whales were assumed to follow a discrete Poisson distribution with a single predicted group size parameter (*λ*) because they were very small,

$$s_{j}\sim Pois\left(\lambda_{\left(s\right)}\right)$$ (3)

whereas group sizes of short-beaked common dolphins were assumed to follow a continuous log-normal distribution, because they were large with a skewed frequency distribution:

$$s_{j}\sim \log N\left(\mu_{\left(s\right)},\sigma_{\left(s\right)}^{2}\right)$$ (4)

A polynomial function with parameters *α* [96] was used to model effective strip half-width as a function of group size and sea state. A normal likelihood with mean 0 was assumed for all random-effects parameters *ε* (*i*.*e*. residuals) [72,74]:

Blue whales:

$$w_{j}=e^{\left(\alpha_{0}+\left(\alpha_{1}\;b_{j}\right)+\left(\alpha_{2}\;s_{j}\right)+\varepsilon_{0}\right)}$$ (5)

Short-beaked common dolphins:

$$w_{j}=e^{\left(\alpha_{0}+\left(\alpha_{1}\;b_{j}\right)+\left(\alpha_{2}\;\ln(s_{j})\right)+\varepsilon_{0}\right)}$$ (6)

The posterior distributions of the parameters *α* were also used for inferring the effective strip half-width in each 1/3°-degree cell (*i*). For that model, we used the effort-weighted Beaufort sea state (*B*) and the predicted mean group sizes as covariates. For blue whales, mean group size was the parameter of the Poisson likelihood (*λ*_*(s)*_) in Eq. 3, whereas for short-beaked common dolphins, mean group size was estimated from the parameters of the log-normal likelihood in Eq. 4, as:

$$\tilde{S}=\mu_{\left(s\right)}+\frac{\sigma_{\left(s\right)}^{2}}{2}$$ (7)

The mean group sizes were fitted using only the sightings’ information. This was done because the procedure of gridding the database into 1/3-degree cells loses information on group size variability.

Blue whales:

$$w_{i}=e^{\left(\alpha_{0}+\left(\alpha_{1}\;B_{i}\right)+\left(\alpha_{2}\;\lambda_{\left(s\right)}\right)+\varepsilon_{1}\right)}$$ (8)

Short-beaked common dolphins:

$$w_{i}=e^{\left(\alpha_{0}+\left(\alpha_{1}\;B_{i}\right)+\left(\alpha_{2}\;\tilde{S}\right)+\varepsilon_{1}\right)}$$ (9)

Also, we did not estimate an independent predicted group size for each cell because there is evidence that dolphin group sizes in the eastern tropical Pacific are highly variable, do not have well-defined spatial patterns, and do not appear to respond to environmental characteristics at the large scales of these surveys [97]. Instead, group size patterns of dolphins in the Northeast Pacific Ocean are more likely to be related to diurnal aggregation dynamics [98], and as such, grouping behavior does not appear to be linked to the species-habitat relationships that we aimed to evaluate from the population density.

Conditional on sea state, group size, and ADT, the number of groups (*n*) observed within each cell *i* was assumed to be Poisson-distributed for both species:

$$n_{i}\sim Pois\left(\lambda_{i}{}_{\left(n\right)}\right)$$ (10)

Since this predicted group counts *λ*_i(n)_ was a key parameter that determined the population density estimations and was affected by both the observational process and the relationship of the species’ with the ADT, we performed a posterior predictive check on its Poisson likelihood, by computing a Bayesian *p*-value, based on the posterior predictive distribution of a goodness-of-fit statistic, in this case, a sums-of-squares type discrepancy [99,100].

The density estimate ($\overset{\wedge }{d}$) was derived from the predicted group counts, since the encounter rate of groups was assumed to depend on the density of animals [32]:

Blue whales:

$$\lambda_{i}{}_{\left(n\right)}=\frac{2\;w_{i}\;L_{i}\;{\hat{d}}_{i}\;\hat{g}(0)}{\lambda_{\left(s\right)}}$$ (11)

Short-beaked common dolphins:

$$\lambda_{i}{}_{\left(n\right)}=\frac{2\;w_{i}\;L_{i}\;{\hat{d}}_{i}\hat{g}(0)}{\tilde{s}}$$ (12)

where *L*_*i*_ was the line-transect effort at cell *i* in kilometers, and $\overset{\wedge }{g}(0)$ was the probability of detecting a group when it was directly on the track line. Previous knowledge on this parameter for both species was included in the model as a beta prior distribution from a conditionally dependent observer design [4], whose parameters *a* and *b* were derived from the mean and the coefficient of variation reported in a previous study [101]. For blue whales *μ*_*g*(0)_ = 0.921 and *CV*_*g*(0)_ = 0.023, whereas for short-beaked common dolphins *μ*_*g*(0)_ = 0.970 and *CV*_*g*(0)_ = 0.017:

$$\hat{g}(0)\sim Beta(a,b)$$ (13)

$$a=c\;\mu_{g(0)}$$ (14)

$$b=c\;\left(1-\mu_{g(0)}\right)$$ (15)

$$c=\left[\frac{\mu_{g(0)}\;\left(1-\mu_{g(0)}\right)}{{\left(\mu_{g(0)}\;CV_{g(0)}\right)}^{2}}\right]-1$$ (16)

The population density in each cell, derived from the group count model (Eqs 11 and 12), was modeled as a log-linear polynomial function of the ADT. For this purpose, the original ADT values were rescaled by subtracting the mean and dividing it by 100, which did not affect the estimations, but improved the Markov Chain Monte Carlo mixing [76]. We tested second-order (*ω* parameters) and third-order (*θ* parameters) polynomials, as well as an average of them using a mixing parameter *ϑ* [102]. Although model selection is still a difficult issue for hierarchical models [103], we used the Deviance Information Criterion (DIC) [104] for choosing the best model among the alternative functions tested. DIC has been criticized when used with complex hierarchical models [105], but it was the only criterion we were able to apply for choosing among these functions:

Second-order polynomial:

$${\hat{d}}_{i(2nd-order)}=e^{\left(\omega_{0}+\left(\omega_{1}\;ADT\right)+\left(\omega_{2}\;ADT^{2}\right)+\varepsilon_{2}\right)}$$ (17)

Third-order polynomial:

$${\hat{d}}_{i(3rd-order)}=e^{\left(\theta_{0}+\left(\theta_{1}\;ADT_{i}\right)+\left(\theta_{2}\;ADT_{i}^{2}\right)+\left(\theta_{3}\;ADT_{i}^{3}\right)+\varepsilon_{2}\right)}$$ (18)

Model average:

$${\hat{d}}_{i(Average)}=\vartheta \;{\hat{d}}_{i(2nd-order)}+(1-\vartheta )\;{\hat{d}}_{i(3rd-order)}$$ (19)

Finally, the quantiles 0.025, 0.975 (*i*.*e*. the limits of the 95-% credible intervals), and the median from the parameter posteriors were used to convert all available original ADT values to inferred population densities to describe their mean spatial distributions and to compare them during El Niño and La Niña years. For this purpose, we assigned a mean value of the Multivariate ENSO Index (MEI) [106,107] to each year, using only values from the period July to December. The MEI was obtained from the Physical Sciences Division of NOAA’s Earth System Research Laboratory (http://www.esrl.noaa.gov/psd/enso/mei/#data).

## Results and Discussion

Blue whales and short-beaked common dolphins are consistently distributed in regions with persistent aggregations of low-trophic-level prey because of their high energetic requirements. At the scale of this study, these aggregations can only occur as a result of major wind-driven upwelling and eddy-like circulation processes. We therefore proposed that the physical structure resulting from these processes is conceptually the factor forcing the long-term distributions of these two cetacean species. Given the high mobility of the animals, we also expected that such spatial distributions would respond relatively rapidly to structural changes in the water column. Other surface environmental predictors, particularly temperature and chlorophyll-*a*, are indirect consequences of the structure produced by the pycnocline dynamics on the upper water column [108] and further, none of those conditions can be interpreted as to be mechanistically linked directly to cetaceans. Instead, they may simply indicate favorable conditions for prey aggregation and/or other biological needs.

From its conception, this was a hypothesis-driven study [109] instead of a data mining process. We wanted to explore the degree to which one variable (ADT) that strongly determines ecosystem structure in the open ocean can be used to make hypothesis-based inferences, in contrast to a common objective in statistical modeling of increasing predictive ability by incorporating multiple environmental variables, even when the exact mechanisms or processes that they drive are unknown. The latter approach can lead to the quandary of having to choose the best model among all possible combinations of environmental predictors available without a strong ecological basis. Given the current difficulties that exist for choosing among complex hierarchical models [74,105,110], it is desirable that predictors in ecological studies be carefully chosen following a clear hypothesis [13,103,111].

The second-order polynomial function (Eq. 17) was selected for both species as the best alternative model of the population density as function of ADT, based on the lowest DIC. The posterior distributions of the three coefficient parameters and the residuals of this function are described in Table 1, as well as the rest of the parameters and statistics used for model evaluation. The assumption that group encounters in cells was Poisson-distributed was critical for evaluating the overall adequacy of the model because it was the parameter that linked the observational process to the ecological process. The mean of the posterior Bayesian *p-*value for evaluating such model assumption was close to 0.5 for both species, indicating good model fitting given the data [99]. The Markov chains’ histories converged and the Monte Carlo (MC) error was very close to 0 for all model parameters (Table 1), indicating that the number of iterations was sufficient to characterize the posterior distributions [89]. As expected, the coefficient parameters that defined the relationship between the population densities and the ADT had the highest MC errors, since ecological processes often involve higher uncertainty than observational ones [112]. There was good convergence of the three chains for all the parameters, as was indicated by $\overset{\wedge }{R}$ statistics close to 1 (Table 1), although the coefficients of the ecological process had the lowest values, also indicating a higher uncertainty.

**Table 1:** Posterior parameter statistics.

Parameter	Symbol	Equations	Mean	SD	Low 2.5%	Median	Hi 97.5%	MC error
**Blue whale**								
Predicted group size	λ_(s)_	3, 8, 11	1.802	0.5659	1.69	1.801	1.918	0.001096
Coefficient parameters of the H-ESW model	α0	5, 8	1.111	0.13790	0.84170	1.112	1.39	0.005304
	α1	5, 8	-0.008098	0.03459	-0.07952	-0.007888	0.05809	0.0010360
	α2	5, 8	0.02194	0.02749	-0.02688	0.02068	0.08075	0.0007942
Bayesian p-value (Poisson likelihood of group counts)	-	10	0.4825	0.49970	-	-	-	0.01016
Detection probability on the transect line	$\hat{g}(0)$	13	0.9207	0.02362	0.86720	0.9234	0.9596	0.0008135
Second-order polynomial coefficient parameters	ω0	17	-9.973	0.2149	-10.42	-9.959	-9.572	0.01782
	ω1	17	-16.2	1.35	-18.98	-16.14	-13.65	0.06797
	ω2	17	-46.88	7.459	-61.32	-47.1	-32.56	0.3933
**Short-beaked common dolphin**								
Mean predicted group size	$\tilde{S}$	7, 12	258.7	12.34	236.4	258.2	284.5	0.2618
Coefficient parameters of the H-ESW model	α0	6	0.1720	0.1141	-0.05129	0.1698	0.4043	0.0066740
	α1	6	-0.1554	0.01904	-0.1932	-0.1555	-0.1161	0.0007586
	α2	6	0.2740	0.02090	0.2328	0.2749	0.3153	0.0012290
Bayesian p-value (Poisson distribution of group counts)	-	-	0.4783	0.49950	0	0	1	0.0113800
Detection probability on the transect line	$\hat{g}(0)$	13	0.9672	0.01959	0.9178	0.9705	0.9937	0.0005652
Second-order polynomial coefficient parameters	ω0	17	-3.1680	0.11460	-3.392	-3.1660	-2.9510	0.0078660
	ω1	17	-10.1400	0.7558	-11.66	-10.1100	-8.6690	0.03139
	ω2	17	-28.9100	5.40600	-40.36	-28.7400	-19.4	0.2927

Only results from the models chosen are shown.

**Fig 2:**
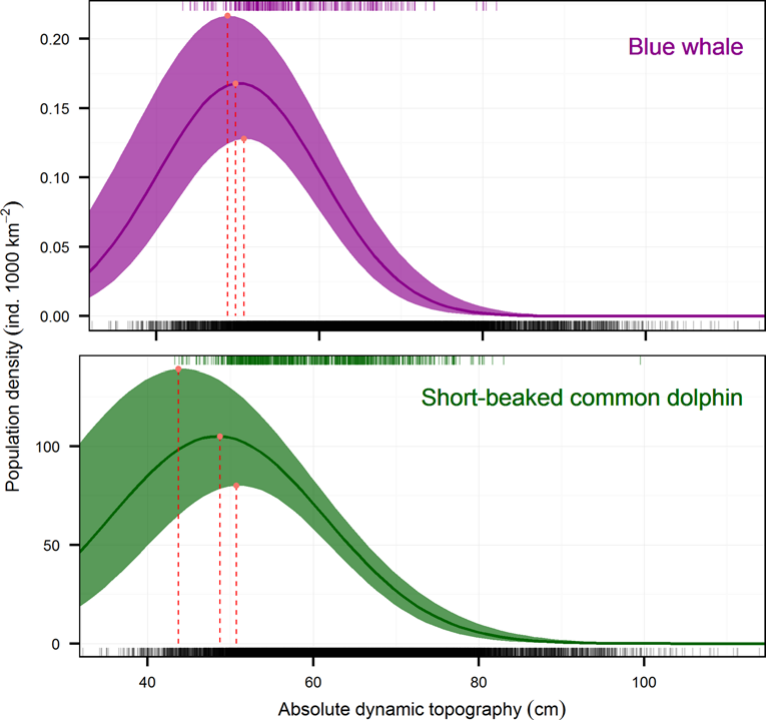
Inferred population densities of blue whales and short-beaked common dolphins in the Northeast Pacific Ocean, as functions of the absolute dynamic topography (ADT) during summer-autumn (July-December). Filled areas represent the 95-% credible intervals. Solid lines represent the median. Black rug lines at the bottom represent values of ADT where there was any survey effort. Color rug lines at the top represent values of ADT where the species were encountered. Red dashed vertical lines represent the optimum habitat in terms of ADT values for each species, where maximum population densities were predicted (red dots) at the lower limit, median, and upper limit of the 95-% credible intervals.

The maximum population densities predicted by our models at the limits of the 95-% credible intervals suggested optimum habitats at ADT values of 48.7 to 50.7 cm for blue whales and 43.7 to 50.7 cm for short-beaked common dolphins (Fig. 2). A decrease in population density at both very low and very high ADT was well supported by the observations, approaching to zero in areas of deep pycnocline (> 80 cm of ADT for blue whales and > 90 cm for short-beaked common dolphins). Predicted densities also decreased in areas of shallow pycnocline that are below the ADT optimum. In the case of blue whales the model predicted a decrease in density at very low ADT values almost as steep as with that of the high values but with higher uncertainty. The decrease in density of short-beaked common dolphins when ADT values were below the optimum was less marked, suggesting a broader habitat preference (Fig. 2). It is intuitive that a deep pycnocline (*i*.*e*. high ADT values) would not be suitable for the aggregation of low-trophic-level prey for blue whales or short-beaked common dolphins, because of the low biological production in these areas, but it is less clear how very low ADT values would also decrease the habitat suitability. This may be related to the well-known fact that the most important aggregations of biomass do not occur at the core of upwelling or eddy-like circulation features, but downstream, where the material can be retained and both phytoplankton and primary consumers can grow and aggregate [113–115]. This has been reported for rorqual whales (Mysticeti: Balaenopteridae) in the Gulf of Saint Lawrence, Canada, where the different species are distributed at particular distances from the core of thermal fronts, according to the relative trophic level of their main prey [116]. This would explain why blue whales have a narrower optimal habitat than short-beaked common dolphins (Fig. 2). Since krill, the primary prey of the blue whale, has relatively limited capacity for horizontal displacement, its distribution is strongly linked to areas of phytoplankton blooms and enhanced biomass retention. In contrast, low-trophic-level fish such as sardine, the main prey of short-beaked common dolphins, have much more mobility [117,118] and can move between and around phytoplankton blooms.

**Fig 3:**
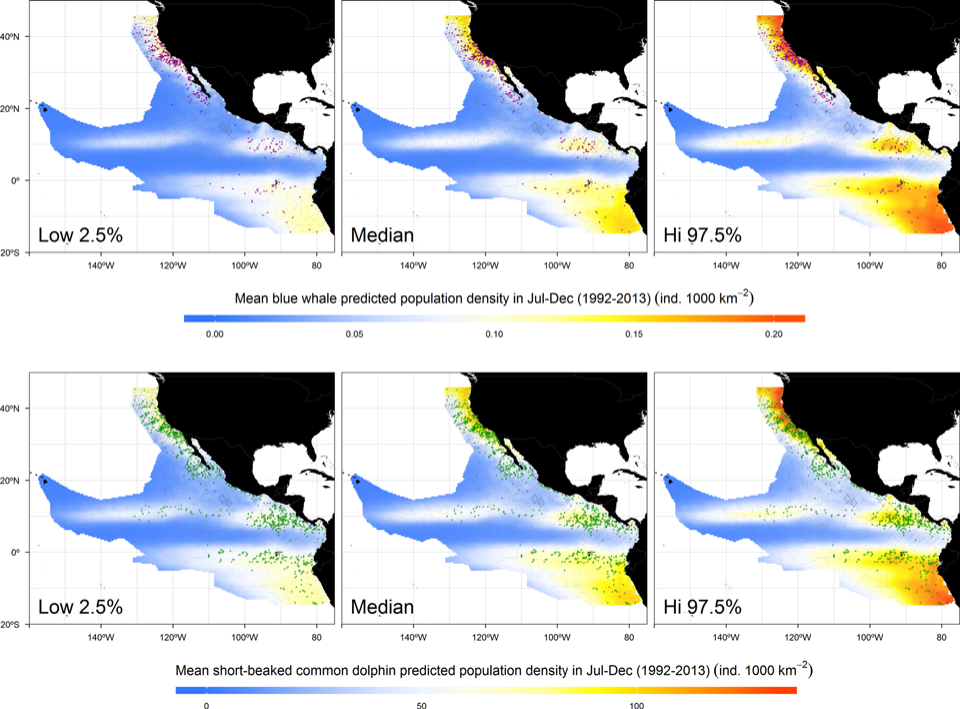
Mean summer-autumn (July-December) distribution of blue whale and short-beaked common dolphin inferred population densities, as functions of the absolute dynamic topography in the Northeast Pacific Ocean. Colored dots represent cells with encounters of the species and gray lines represent the survey effort.

Our results predicted densities above the mean for both species in the central area of the Northeast Pacific Ocean, especially in the Costa Rica Dome and the North Equatorial Countercurrent thermocline ridge, and suggested a broader range of distribution for the short-beaked common dolphin, compared to that of the blue whale in those areas. Nevertheless, the California Current System, the Humboldt Current System, and the Equatorial Cold Tongue remained as the most important habitats for both species (Fig. 3). The latter is especially important for short-beaked common dolphins. In the California Current System, blue whales were predicted nearest to the coast while short-beaked common dolphins were distributed in more oceanic waters. This was in agreement with modeled distributions predicted by previous studies [35,119]. Likewise, in the Frontal System off Baja California, high densities of blue whales were predicted in a narrow corridor compared to the area that would be occupied by short-beaked common dolphins (Fig. 3). Since krill has a lower position in the trophic web compared to that of low-trophic-level fish, it was expected that blue whales would occur closer to the thermal fronts than short-beaked common dolphins [116]. In the Gulf of California, the model predicted low densities of blue whales, which agrees with the well-known summer-fall migration to the feeding grounds in the California Current System and farther to the north [19,65]. Only few blue whales have been recorded during summer-autumn in the northern Gulf of California, around *Canal de Ballenas* [120,121], which is where our model predicted the highest densities of both species. In the Costa Rica Dome, blue whales seemed to be distributed in high densities closer to the Dome’s core, whereas short-beaked common dolphins occupied practically the entire area under the Dome’s influence (see Figs. 1 and 3), and extending into the Gulf of Panama and off Colombia, where blue whales were virtually absent.

Given the observed and modeled distributions, the North Equatorial Countercurrent thermocline ridge was an important habitat for short-beaked common dolphins, but less so for blue whales. Despite the model’s prediction of slightly above average blue whale densities within this feature (Fig. 3), there were no sightings of the species during the surveys. However, observations from other studies reporting blue whales [36,65] as well as large krill aggregations [49] in the North Equatorial Countercurrent thermocline ridge support our model predictions of suitable habitat within this feature. As with the Costa Rica Dome, we do not know if blue whales visiting the North Equatorial Countercurrent thermocline ridge come only from the Northern Hemisphere [65], or if there are some animals using this system year-round [24]. Habitat models based on the routes revealed by satellite-tagged individuals that have been tracked to this remote area [65] would help address this gap in knowledge.

**Fig 4:**
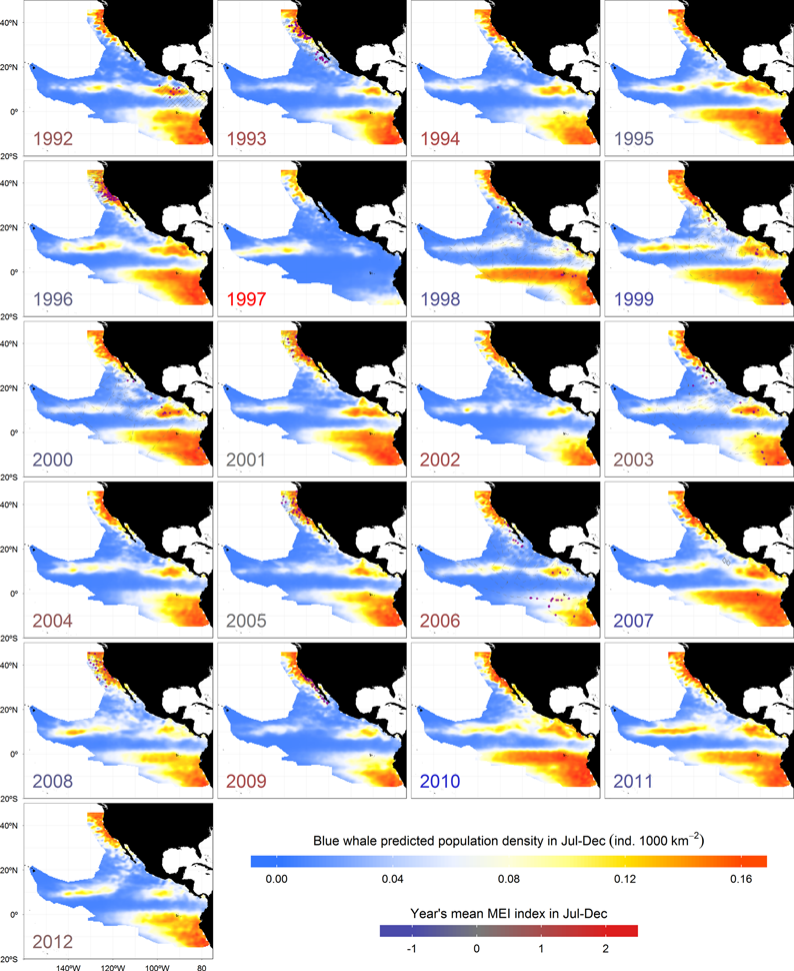
Inter-annual spatial fluctuations of blue whale population densities, inferred from ADT during summer-autumn (July-December). Each year label has a color according to the mean Multivariate ENSO Index (MEI) during that period. Predictions in years with effort are based only in mean ADT values from the months surveyed. The rest are from July-to-December means. Magenta dots represent sightings and gray lines represent the survey effort.

**Fig 5:**
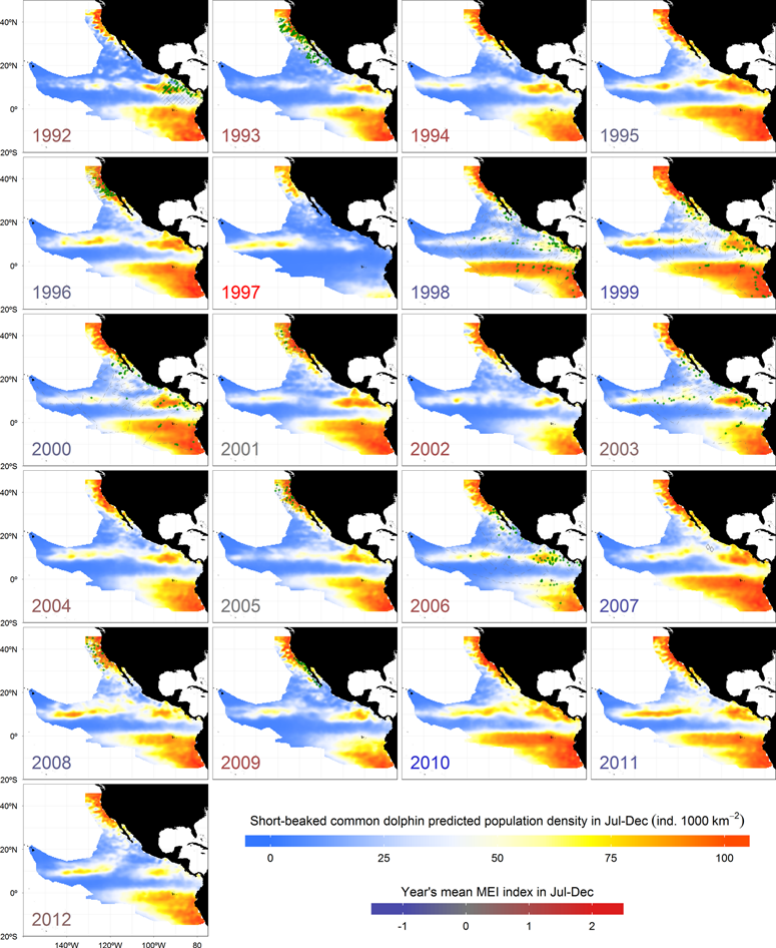
Inter-annual spatial fluctuations of short-beaked common dolphin population densities, inferred from ADT during summer-autumn (July-December). Each year label has a color according to the mean Multivariate ENSO Index (MEI) during that period. Predictions in years with effort are based only on mean ADT values from the months surveyed. The rest are from July-to-December means. Magenta dots represent sightings and gray lines the survey effort.

The latter result of blue whale predictions in an area where there were no sightings during the surveys underscores the issue of the biases in the detections and of survey coverage, both in time and space. Field observations were limited by budget and weather conditions, and the surveys did not cover evenly the study area. Instead, they were intended to cover particular strata in each year. Although this was certainly a limitation in data coverage, our habitat-based estimates of population density reduced the effect of such spatial heterogeneity in search effort by introducing information on the probability that the species was present, regardless of whether it was detected or not [76]. The lack of sightings during the surveys could be attributed to insufficient effort to detect the species, to effort under conditions of low detectability, or to habitat that was insufficiently suitable to produce a detectable spatial increase in the population density at the time the surveys were made [76] (Figs. 4 and 5).

The North Equatorial Countercurrent thermocline ridge as potential habitat for blue whales was not the only case in our results that differed from the observations. The models suggested high densities of both species at the high latitude ends of the study area in the California Current and the Humboldt Current Systems, where there were few encounters of the species. Low effort coverage may explain the few observations in the Humboldt Current System but not in the California Current System. Instead, the observations strongly indicate that the species have diminished occurrence at those latitudes, which can be interpreted as a spatial boundary for their distribution. In this case, what the models are likely suggesting is that the physical habitat continues to be suitable for both species in these areas, but that there could be other eco-physiological factors restricting their occurrence. For example, differences in light penetration and heat balance at high latitudes may lead to the development of different types of low-trophic-level prey [49,122], attracting other cetacean species better adapted to forage on them. We note, however, that while most of the blue whales in the Northeast Pacific Ocean feed in the California Current System [123], they also feed at higher latitudes in waters off Oregon and Washington [65,124] as well as in the Alaska Gyre and around the Aleutian Islands [17].

There was also some disparity between the few observations and the model results in the northern Humboldt Current System. It is important to clarify that blue whales from the Southern Hemisphere are a different population from those of the north [22] and that their numbers are still low because they are in a process of slow recovery form whaling that drove them to near extinction [82]. They migrate to mid and low latitudes during the summer-autumn of the Northern Hemisphere (*i*.*e*. winter-spring of the Southern Hemisphere) to reproduce and feed [21]. Therefore, we would expect that the higher whale densities during this period are concentrated in the South Equatorial Countercurrent and the northern Humboldt Current System, where higher temperatures would be more suitable for calving and where prey aggregations remain high. Also, similar to the North Equatorial Countercurrent thermocline ridge, a very low survey effort in this area could lead to a low number of sightings.

For short-beaked common dolphins, there are almost no sightings despite the intensive survey effort in the northern California Current System. The most extreme geographic records of the species coincide with the limits of the sardine distribution [122]. This latitudinal limit could be driven by competition with other species better adapted to exploit different types of low-trophic-level fish and squid in those areas, such as the Pacific white-sided dolphin (*Lagenorhynchus obliquidens*), whose size and diet are similar to those of the short-beaked common dolphins [1]. This result exemplifies why we should not expect ADT, or any other habitat variable for that matter, to predict a species’ distribution faithfully, especially outside the limits of a study area, where additional ecological knowledge is necessary to understand failures in the models.

Predicted densities for both species responded negatively to strong El Niño anomalies in all regions (Figs. 4 and 5), but especially in the South Equatorial Countercurrent, which is directly influenced by downwelling Kelvin waves that deepen the pycnocline. The model suggested that both species would have abandoned that region during the extreme 1997 El Niño in response to the generalized increase of ADT (*i*.*e*. pycnocline deepening and enhanced stratification), restricting their distribution to the core of the remaining productive habitats. The cores of the Frontal System off Baja California and the Costa Rica Dome remained with densities slightly above the mean, as did the northern Gulf of California, which has been described as a refuge for rorqual whales during El Niño anomalies [120]. The species also appeared to concentrate in the central and northern California Current System, and in the North Equatorial Countercurrent thermocline ridge, where the pycnocline does not deepen as much. These results suggest that ENSO anomalies can reduce or expand the extent of favorable foraging habitats for blue whales and short-beaked common dolphins. The concentration of short-beaked common dolphins off central California in response to the 1997 El Niño has already been described [125]. We can also speculate that blue whales migrating from high latitudes in the Southern Hemisphere might not be able to forage successfully in the South Equatorial Countercurrent and the northern Humboldt Current System during extreme El Niño years, being forced to remain further to the south. Unfortunately, there were no surveys during the strong 1997 El Niño to compare with the model predictions. Nevertheless, the spatial distribution of the observations during more moderate El Niño anomalies in 1992, 1993, and 2006 agrees with the model results for both species (Figs. 4 and 5).

The reduced spatial coverage of the predicted population densities during 1997 El Niño, the strongest in the last 20 years, suggest that the population numbers of both species could have declined dramatically. Although such decrease in habitat availability would result in a re-distribution of the populations to areas remaining highly productive (even outside our study area, as already discussed), we cannot underestimate the potential of those phenomena to affect considerably the survival of species with high energetic requirements, especially for new born calves or weak adults. Based on our results of the redistribution of blue whales and short-beaked common dolphins during El Niño events, and the corresponding decrease in population density in several areas of the Northeast Pacific Ocean, long-term negative effects on their populations might be expected if there is an increase in the frequency and intensity of such events arising from climate change, as some studies have suggested [126–128]. In the case of La Niña anomalies, the density responses were not as dramatic as those described for El Niño (Figs. 4 and 5). Both species had similar distributions and population density values to those of the mean conditions (Fig. 3). This is because the magnitude of pycnocline shoaling during La Niña is not equivalent to its deepening during El Niño [71,129]. The processes driving these phenomena are not linearly related and we cannot expect them to have equal but opposite effects, either physical or biological [130].

The consistent relationship between the population density of cetaceans with environmental conditions measured by satellite sensors allow for broad-scale predictions of abundance and open the possibility of forecasting population trends and distribution into the future [35]. This is especially true for ADT, for which near-real-time, global products are readily available. The availability of high-quality altimetry products is particularly appealing for applications involving proposals for spatially explicit conservation areas for species whose distributions have clear associations with the structure of the water column. Nevertheless, our results also indicate that model predictions are only reliable in areas where adequate spatio-temporal survey coverage is available to derive robust relationships and where sufficient ecosystem information exists to facilitate interpretation.

## Supporting Information

S1 Fig Frequency distributions of the main variables used. (TIFF)

S1 Text Code of models in the BUGS language. Only the models with the lowest Deviance Information Criteria are shown.
(DOCX)
