# Supplementary figures and images for: Inferring Cetacean Population Densities from the Absolute Dynamic Topography of the Ocean in a Hierarchical Bayesian Framework
Source: PLoS One. 2015 Mar 18;10(3):e0120727. doi: 10.1371/journal.pone.0120727 (PMC4364891; doi:10.1371/journal.pone.0120727)

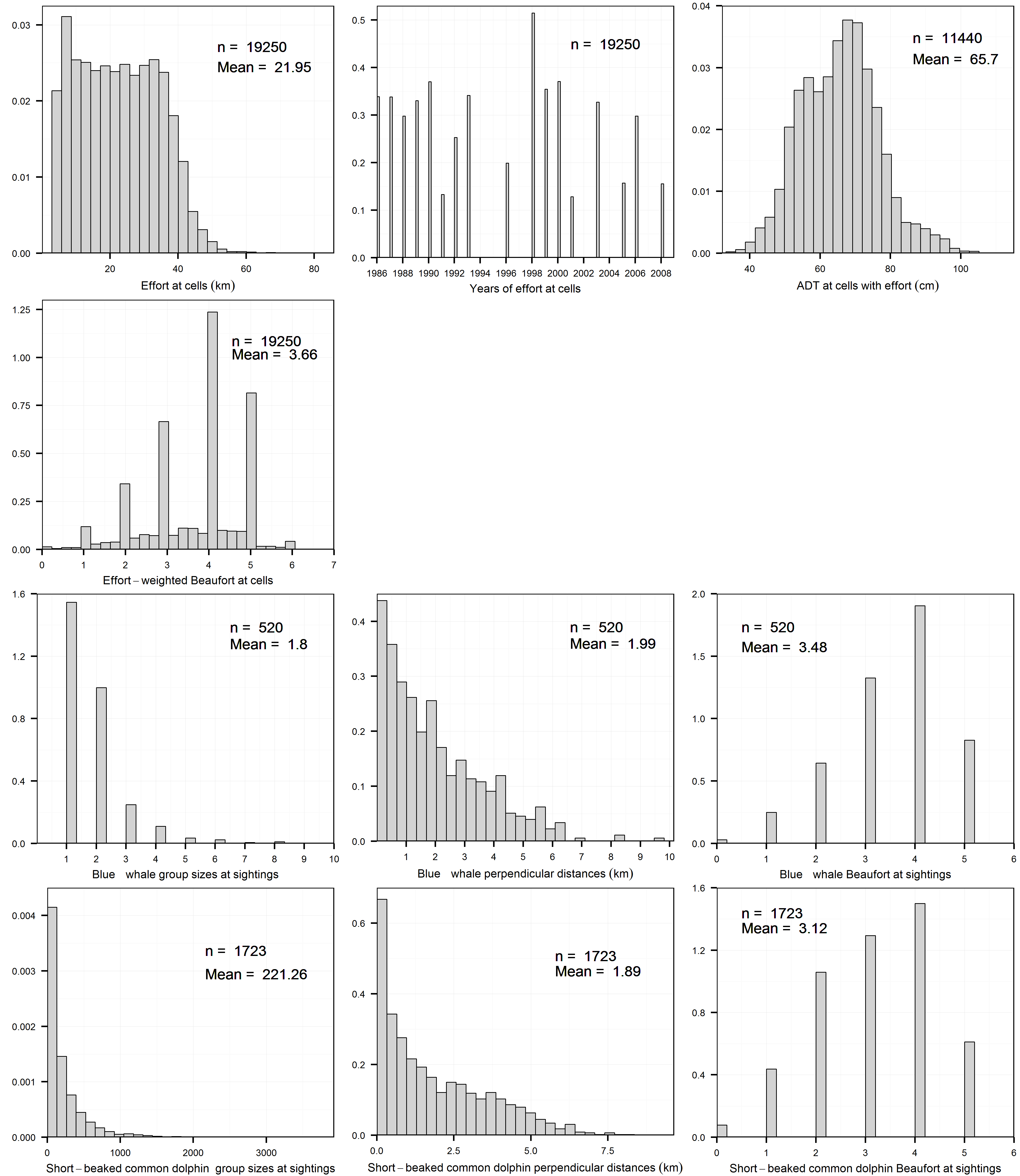

Supplement: S1 Fig — (TIFF) [file pone.0120727.s001.tiff]
