# Supplementary material for: Inferring Cetacean Population Densities from the Absolute Dynamic Topography of the Ocean in a Hierarchical Bayesian Framework
Source: PLoS One. 2015 Mar 18;10(3):e0120727. doi: 10.1371/journal.pone.0120727 (PMC4364891; doi:10.1371/journal.pone.0120727)
Supplement: S1 Text — Only the models with the lowest Deviance Information Criteria are shown. (DOCX) [file pone.0120727.s002.docx]

***##### Short-beaked common dolphins: #####***

##### PRIORS: #####

a0 ~ dunif(-0.2,0.6)

a1 ~ dunif(-0.3,-0.02)

a2 ~ dunif(0.05,0.42)

sd.eps.a ~ dunif(0.15,0.65)

sd.eps.a2 ~ dunif(0.05,0.42)

logmu.gs ~ dunif(3.5,5.5)

logsd.gs ~ dunif(8.5,13.5)

nu0 ~ dunif(-3.6,-2.1)

nu1 ~ dunif(-14,-7.5)

nu2 ~ dunif(-45,-18)

sd.eps.nu ~ dunif(1.12,1.95)

#### OBSERVATION MODELS ####

## Models from sightings (j): ##

logvar.gs <- log(1+pow(logsd.gs/logmu.gs,2))

logtau.gs <- 1/logvar.gs

eps.a.tau <- 1/(sd.eps.a*sd.eps.a)

for (j in 1:1723){

perp.dist.sigh[j] ~ dnorm(0,perp.dist.tau[j]) **# Eq. 1**

perp.dist.tau[j] <- 1/(pow(w.sigh[j],2)*(2/pi)) **# Eq. 2**

gp.sz.sigh[j] ~ dlnorm(logmu.gs,logtau.gs) **# Eq. 4**

eps.a[j] ~ dnorm(0,eps.a.tau)

w.sigh[j] <- exp(a0+(a1*beauf.sigh[j])+(a2*log(gp.sz.sigh[j]))+eps.a[j]) **# Eq. 6**

}

mean.gs <- exp(logmu.gs+0.5*logvar.gs) **# Eq. 7**

#### g(0): ####

g0.barlow ~ dbeta(102.8362,3.180502) **# Eqs. 13 to 16**

#### Models from cells (i): ####

eps.a2.tau <- 1/(sd.eps.a2*sd.eps.a2)

eps.nu.tau <- 1/(sd.eps.nu*sd.eps.nu)

for (i in 1:11173)

#### Effective strip half-width model for cells: ####

eps.a2[i] ~ dnorm(0,eps.a2.tau)

w.cell[i] <-exp(a0+(a1*beauf.cell[i])+(a2*log(mean.gs))+eps.a2[i]) **# Eq. 9**

## Predicted group counts: ##

n.groups.cell[i] ~ dpois(pred.gp[i]) **# Eq. 10**

pred.gp[i] <- (2*w.cell[i]*eff.cell[i]*dens.cell[i]*g0.barlow)/mean.gs **# Eq. 12**

#### Check the group counts likelihood: ####

squared.res.obs.gp.counts[i] <- pow(n.groups.cell[i]-pred.gp[i],2)

new.n.groups.cell[i] ~ dpois(pred.gp[i])

new.squared.res.gp.counts[i] <- pow(new.n.groups.cell[i] - pred.gp[i], 2)

###################################### ECOLOGICAL MODEL: ######################################

eps.nu[i] ~ dnorm(0,eps.nu.tau)

dens.cell[i] <- exp(nu0+(nu1*ssh.cells[i])+(nu2*pow(ssh.cells[i],2))+eps.nu[i]) **# Eq. 17**

}

#### POSTERIOR PREDICTIVE CHECK: ####

fit.obs.counts <- sum(squared.res.obs.gp.counts[])

fit.new.counts <- sum(new.squared.res.gp.counts[])

test.fit.counts <- step(fit.new.counts-fit.obs.counts)

b.p.value.counts <- mean(test.fit.counts) # Bayesian p-value

***##### Blue whales: #####***

#### PRIORS: ####

a0 ~ dunif(0.4,1.6)

a1 ~ dunif(-0.16,1)

a2 ~ dunif(-1,0.15)

sd.eps.a ~ dunif(0.05,0.8)

sd.eps.a2 ~ dunif(0.15,0.7)

pred.gp.size ~ dunif(1,3)

nu0 ~ dunif(-13,-7)

nu1 ~ dunif(-21,-9)

nu2 ~ dunif(-65,-30)

sd.eps.nu ~ dunif(1,3)

#### OBSERVATION MODELS ####

## Models from sightings (j): ##

eps.a.tau <- 1/(sd.eps.a*sd.eps.a)

for (j in 1:1723){

perp.dist.sigh[j] ~ dnorm(0,perp.dist.tau[j]) **# Eq. 1**

perp.dist.tau[j] <- 1/(pow(w.sigh[j],2)*(2/pi)) **# Eq. 2**

gp.sz.sigh[j] ~ dpois(pred.gp.size) **# Eq. 3**

eps.a[j] ~ dnorm(0,eps.a.tau)

w.sigh[j] <- exp(a0+(a1*beauf.sigh[j])+(a2*gp.sz.sigh[j])+eps.a[j]) **# Eq. 5**

}

#### g(0): ####

g0.barlow ~ dbeta(148.4174,12.7307) **# Eqs. 13 to 16**

#### Models from cells (i): ####

eps.a2.tau <- 1/(sd.eps.a2*sd.eps.a2)

eps.nu.tau <- 1/(sd.eps.nu*sd.eps.nu)

for (i in 1:11440)

#### Effective strip half-width model for cells: ####

eps.a2[i] ~ dnorm(0,eps.a2.tau)

w.cell[i] <-exp(a0 +(a1 *beauf.cell[i])+(a2* pred.gp.size)+eps.a2[i]) **# Eq. 8**

## Predicted group counts: ##

n.groups.cell[i] ~ dpois(pred.gp[i]) **# Eq. 10**

pred.gp[i] <- (2*w.cell[i]*eff.cell[i]*dens.cell[i]*g0.barlow)/mean.gs **# Eq. 12**

## Check the group counts likelihood: ##

squared.res.obs.gp.counts[i] <- pow(n.groups.cell[i]-pred.gp[i],2)

new.n.groups.cell[i] ~ dpois(pred.gp[i])

new.squared.res.gp.counts[i] <- pow(new.n.groups.cell[i]-pred.gp[i], 2)

#### ECOLOGICAL MODEL: ####

eps.nu[i] ~ dnorm(0,eps.nu.tau)

dens.cell[i] <- exp(nu0+(nu1*ssh.cells[i])+(nu2*pow(ssh.cells[i],2))+eps.nu[i]) **# Eq. 17**

}

#### POSTERIOR PREDICTIVE CHECK: ####

fit.obs.counts <- sum(squared.res.obs.gp.counts[])

fit.new.counts <- sum(new.squared.res.gp.counts[])

test.fit.counts <- step(fit.new.counts-fit.obs.counts)

b.p.value.counts <- mean(test.fit.counts) # Bayesian p-value
